# Metabolic Enzymes Enjoying New Partnerships as RNA-Binding Proteins

**DOI:** 10.1016/j.tem.2015.09.012

**Published:** 2015-12

**Authors:** Alfredo Castello, Matthias W. Hentze, Thomas Preiss

**Affiliations:** 1 Department of Biochemistry, University of Oxford, South Parks Road, Oxford OX1 3QU, UK; 2 European Molecular Biology Laboratory, Meyerhofstrasse 1, 69117 Heidelberg, Germany; 3 EMBL–Australia Collaborating Group, Department of Genome Sciences, The John Curtin School of Medical Research, The Australian National University, Acton (Canberra), ACT 2601, Australia; 4 Victor Chang Cardiac Research Institute, Darlinghurst (Sydney), New South Wales 2010, Australia

**Keywords:** metabolic enzymes, metabolon, RNA, RNA-binding proteins, post-transcriptional regulation, post-translational modifications

## Abstract

In the past century, few areas of biology advanced as much as our understanding of the pathways of intermediary metabolism. Initially considered unimportant in terms of gene regulation, crucial cellular fate changes, cell differentiation, or malignant transformation are now known to involve ‘metabolic remodeling’ with profound changes in the expression of many metabolic enzyme genes. This review focuses on the recent identification of RNA-binding activity of numerous metabolic enzymes. We discuss possible roles of this unexpected second activity in feedback gene regulation (‘moonlighting’) and/or in the control of enzymatic function. We also consider how metabolism-driven post-translational modifications could regulate enzyme–RNA interactions. Thus, RNA emerges as a new partner of metabolic enzymes with far-reaching possible consequences to be unraveled in the future.

## Trends

Genetic control of metabolism is currently best understood at the level of transcription and epigenetics. Only limited information is available on post-transcriptional regulation of metabolism.

While a few metabolic enzymes were previously known to moonlight as RNA-binding proteins in physiologically relevant contexts, recent discoveries highlight that several dozen of metabolic enzymes belonging to a wide spectrum of pathways exhibit RNA-binding activity in living mammalian cells.

Abundant RNA–enzyme interactions might suggest novel roles of RNA in affecting enzyme function, for instance, as competitive inhibitors or allosteric regulators. A function of RNA as assembly scaffold for enzyme complexes is also conceivable, with potentially wide-ranging implications for our understanding of how cells organize and control metabolic flux. Finally, enzymes can moonlight as regulators of (m)RNAs, as exemplified by aconitase/IRP1 and GAPDH.

## Regulation of Metabolic Networks

Metabolic enzymes were long considered to be constitutively expressed housekeeping proteins, and even nowadays glyceraldehyde-3-phosphate dehydrogenase (GAPDH) mRNA continues to be broadly used for normalization of real-time quantitative PCR experiments. However, this traditional view is challenged by advances in many areas, including developmental, cancer, and stem cell biology. The expression profiles of metabolic enzymes are controlled by cell identity, which enables tissue metabolic specialization. Furthermore, metabolic enzyme expression is also subject to fine-tuning temporal regulation in response to feast/famine and to day/night cycles (reviewed in 1, 2, respectively).

The discovery of the nuclear hormone receptors (NHRs) in the 1980s represented a breakthrough in the understanding of the transcriptional control of metabolic networks. NHRs represent an extended family of ligand-responsive DNA-binding proteins that, upon activation, can switch transcriptional programs in cooperation with coactivators or corepressors [3]. NHRs are transcriptional master regulators of metabolism by altering the metabolic enzyme profiles in response to feeding and fasting as well as circadian signaling. An illustrative example is the role of NHRs in liver metabolism. Secretion of cortisol from the adrenal gland during prolonged starvation induces the activation of the glucocorticoid receptor in the liver. This leads to the transcription of two master regulators of sugar metabolism, glucose-6-phosphatase (G6PC) and phosphoenolpyruvate carboxykinase (PECK), which promote the synthesis of glucose via gluconeogenesis 1, 4. By contrast, liver X receptors (LXRs) and farnesoid X receptor (FXR) are activated by feeding-induced synthesis of their respective ligands, oxysterols and bile acid. In antagonism to fasting-activated NHRs, both LXRs and FXR suppress gluconeogenesis by upregulating the expression of glucokinase, which promotes glucose utilization, and by increasing glycogen synthesis 5, 6, 7. LXR activation also leads to an enhancement of triacylglycerol synthesis by upregulating the genes involved in lipogenesis [8]. Thus, the study of transcription factors such as NHRs and numerous others has contributed much to our understanding of the genetic control of metabolism 1, 2, 3, 9, 10.

Importantly, transcriptomes only partially correlate with their corresponding proteomes, implying that RNA-based post-transcriptional regulation should play an important role in sculpting cellular proteomes [11]. Interestingly, a few metabolic enzymes had been noted to bind RNA themselves and, in some instances, participate in the post-transcriptional control of specific mRNAs [12]. For example, thymidine synthase (TYMS) can bind and inhibit the translation of its own RNA when the levels of its substrates are low, establishing a negative feedback loop 13, 14, 15. Conceptually, such a mechanism represents a simple yet effective way to adjust to conditions when the enzyme is not required. In this review, we discuss the emerging roles of protein–RNA interactions in controlling metabolism.

## Moonlighting Enzymes: Findings from RNA Interactomes

Over the past three decades, sporadic reports have shown that metabolic enzymes can moonlight as RNA-binding proteins (RBPs) and, in some instances, regulate the expression of their target mRNAs 12, 16, 17 (Table 1). These **moonlighting enzymes** (see Glossary) participate in varied metabolic pathways, such as glycolysis, the tricarboxylic acid (TCA) cycle, lipid metabolism, and deoxynucleotide biosynthesis, and catalyze different reactions. In most cases, RNA binding was observed *in vitro*, using filter binding or electrophoretic mobility shift assays 12, 15, 18, 19, 20. While most of the reported moonlighting metabolic enzymes still await validation in living cells and animals, the functions and modes of RNA binding of aconitase 1 (ACO1, also known as iron regulatory protein 1, IRP1), GAPDH, and TYMS have been explored by biophysical and structural approaches [21], and investigated in cellular and animal models as described later 22, 23, 24, 25. Insights from these examples form the basis of the ‘**REM (RNA–enzyme–metabolite) hypothesis**’, which proposes the existence of regulatory links between gene expression and intermediary metabolism mediated by moonlighting RNA-binding metabolic enzymes [17].

Recent system-wide approaches have been developed to identify a (near) complete compendium of RBPs. Initially, two parallel works used *Saccharomyces cerevisiae* proteome-wide protein arrays to interrogate protein binding to RNA *in vitro*. These studies catalogued 180 [26] and 42 proteins [27], respectively, as putative RBPs, including many not previously known to interact with RNA. Among the dozen metabolic enzymes reliably associated with RNA *in vitro*, oxidoreductases and proteins involved in lipid metabolism were the most prominent classes of putative moonlighting metabolic enzymes. The peroxisomal malate dehydrogenase (MDH3) was identified in both studies as an RBP; immunoprecipitation followed by microarray (RIP-Chip) showed modest RNA-binding capacity towards a limited pool of target RNAs [26]. Because the peroxisome is not an organelle classically associated with RNA biology, these results called for further experimental validation in cellular models.

**Figure 1:**
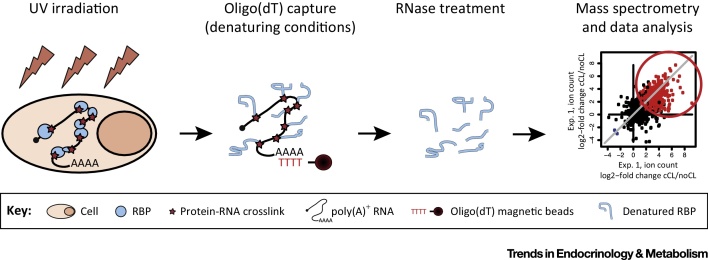
Schematic Representation of RNA Interactome Capture. Living cell monolayers are irradiated with UV light to covalently link direct protein–RNA interactions. Polyadenylated RNA and covalently bound protein partners are isolated by oligo(dT) pull-down under denaturing conditions. After RNase treatment, the RNA-binding protein (RBP) repertoire is determined by quantitative mass spectrometry, comparing proteins isolated from crosslinked cells (cCL) with those present in a mock pull-down (noCL). Only proteins with consistent enrichment across replicates (encircled in red) are considered as the RNA interactome.

To address the technical limitations of *in vitro* RBP identification screens, two groups developed in parallel a new approach named **RNA interactome capture** (Figure 1). Applying UV crosslinking to proliferative cell monolayers, followed by stringent denaturing oligo(dT) isolation of protein–RNA complexes and quantitative mass spectrometry, these studies identified a total of 1106 high-confidence RBPs in HeLa and HEK293 cells 28, 29. Notably, hundreds of them were novel RNA interactors and lacked known RNA-binding domains (RBDs). This method offers several advantages over previous approaches: (i) UV light promotes free radical formation at the nucleotide base that can establish covalent bonds only with amino acids placed at ‘zero distance’ (≤2 Å); (ii) UV crosslinking does not promote protein–protein crosslinks; (iii) because UV is applied directly to living cells, hybridization with oligo(dT) captures native protein–RNA complexes; (iv) nucleic acid hybridization is compatible with high salt and denaturing agents including chaotropic detergents, thus allowing stringent removal of noncovalent binders; and (v) to qualify as high-confidence RBP, quantitative information and rigorous statistic methods are applied 29, 30.

Among the newly identified RBP classes, the RNA interactome studies reported 23 distinct metabolic enzymes associated with polyadenylated RNAs 28, 29, 31 (Table 1), suggesting that the interplay between RNA and metabolism is broader than previously realized and supporting the REM network hypothesis. Among these moonlighters, aldolase and trifunctional enzyme subunit β (HADHB) had previously been recognized to bind RNA *in vitro* [12] and the interaction of enolase 1 (ENO1), hydroxymethyltransferase (SHMT1), and pyruvate kinase M2 (PKM2) with RNA was validated in cells by an independent approach 29, 30. Applying UV crosslinking, immunoprecipitation, and RNA sequencing (CLIPseq), it was shown that ENO1 and SHMT2 associate with hundreds of different mRNAs in HeLa cells, but display distinct binding patterns from each other, suggesting selectivity of binding [29]. In agreement, bacterial enolase has been recently identified, together with the RNase E, as a part of the degradosome complex, which suggests that the relationship of this enzyme with RNA is already observable in prokaryotes [32]. Although the 23 moonlighting enzymes identified by the RNA interactome studies belong to different metabolic pathways and catalyze distinct reactions, 13 of them bind either dinucleotides or mononucleotides (Table 1). This suggests that protein domains commonly involved in nucleotide binding, such as the Rossmann fold, may represent suitable protein surfaces to interact with RNA, as discussed in more detail later.

Interestingly, some of the already known and newly discovered moonlighting RBPs are linked to hereditary diseases. Mutations in inosine 5′-monophosphate dehydrogenase 1 (IMPDH1), a dual RNA-binding and dinucleotide-binding enzyme [33], cause retinitis pigmentosa [34], an eye disease with severe vision impairment attributable to the progressive degeneration of the photoreceptors in the retina [35]. Importantly, the disease-associated D226N IMPDH mutant exhibits metabolic activity but it is unable to bind nucleic acids [36]. IMPDH is involved in the post-transcriptional regulation of rhodopsin mRNA and this disease-associated mutation reduces its association with polysomes and thus its translation efficiency [37]. Retinitis pigmentosa is also caused by mutations in components of the splicing machinery, such as U4/U6 small nuclear ribonucleoprotein Prp3 (PRPF3), PRPF8, and PRPF31, suggesting a considerable role of RNA biology in this disease [38].

The mitochondrial enzyme 17β-hydroxysteroid dehydrogenase 10 (HSD17B10; also known as MRPP2), catalyzes the dehydrogenation of 17-hydroxysteroids in steroidogenesis. However, it was catalogued as an RBP in HEK293 cells [28] and also moonlights as a component of mitochondrial ribonuclease P, which is involved in the processing of the mitochondrial tRNAs [39]. HSD17B10 deficiency causes neurodegeneration in humans and has been associated with Alzheimer's disease. Curiously, there is no correlation between the degree of catalytic activity of the disease-associated mutant enzymes and the severity of the disease [40], suggesting that the molecular mechanism underlying this pathology does not primarily derive from the catalytic activity of HSD17B10. Indeed, a recent study revealed that knock-down or mutation of HSD17D10 induces a defect in the processing of the heavy strand of the mitochondrial RNA [41]. In summary, abrogation of the RNA-related function of these moonlighting metabolic enzymes is associated with phenotypic consequences, supporting the importance of these protein–RNA interactions in cell biology.

## The IRP1/Aconitase Paradigm

**Figure 2:**
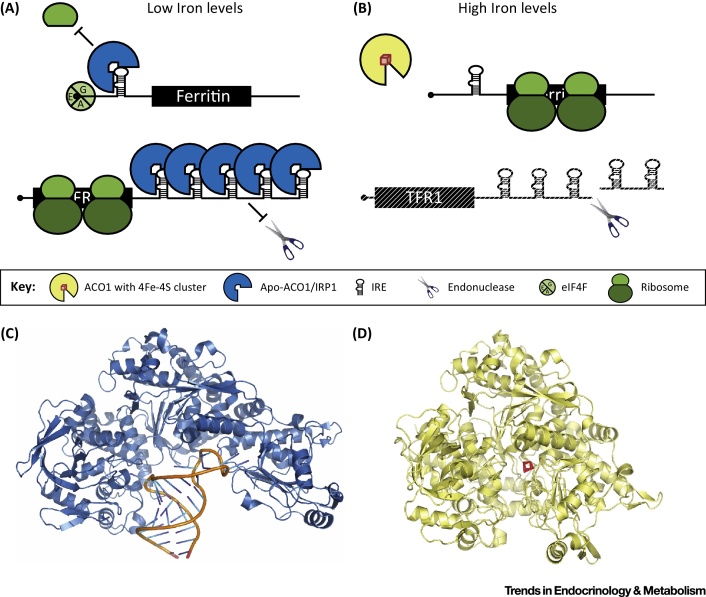
Iron Regulatory Protein 1 (IRP1) Functions as Cytosolic Aconitase and RNA-Binding Protein (RBP). (A) IRP1 binds to several mRNAs when not assembled with the 4Fe–4S cluster due to low intracellular concentrations of iron. Among the best-studied examples is the binding of IRP1 to the iron-responsive element (IRE) in the 5′ untranslated region (UTR) of the ferritin mRNA to repress its translation. Since this mRNA encodes an iron-storage protein, diminished ferritin levels will promote an increase of free iron. Conversely, IRP1 increases the stability of transferrin receptor mRNA when binding to IREs in its 3′ UTR. An increase in transferrin receptor levels will promote cellular iron uptake. (B) Conversely, when IRP1 bears a 4Fe–4S cluster due to high intracellular iron concentration, it becomes active as cytosolic aconitase, catalyzing the interconversion between citrate and isocitrate. (C) Ribbon diagram of IRP1 bound to an IRE (PDB 3SNP). (D) Ribbon diagram of IRP1 crystalized as aconitase with the active site 4Fe–4S cluster (shown in red) (PDB 2B3Y).

In the early 1990s, it became clear that the RBP intensively studied for its role in the regulation of cellular iron metabolism, iron regulatory protein (IRP) 1, is identical with cytosolic aconitase 42, 43, 44, 45. The role of IRP1 in the post-transcriptional control of iron homeostasis illustrates the important biological role that RNA-binding enzymes may play *in vivo*. RNA stem loop structures termed iron-responsive elements (IREs) were first found in the 5′ untranslated regions (UTRs) of ferritin mRNAs [46] and in the 3′ UTR of transferrin receptor mRNA 47, 48 (Figure 2). Specific IRE-binding proteins were identified 49, 50 and later termed iron regulatory protein 1 (IRP1) [51] and IRP2 52, 53. IRE motifs have since been found in other mRNAs, mostly encoding proteins involved in iron homeostasis and utilization, and the mechanisms by which IRPs regulate these targets have been elucidated [54]. Specifically, IRPs bind to RNAs in iron-deficient cells, and interaction with an IRE in the 5′ UTR blocks mRNA translation, while binding to IREs in the 3′ UTR leads to mRNA stabilization; in this way, the IRPs are crucial to maintaining appropriate intracellular iron levels (Figure 2A,B). Both proteins are broadly expressed across tissues and single knockout mice are viable, while simultaneous knockout of both IRPs is early embryonic lethal, indicating essential but largely redundant functions. Nevertheless, the single knockout phenotypes also demonstrate specific roles for IRP1 in erythropoiesis and the cardiovascular system, while IRP2 is of particular importance in erythroblasts and the nervous system (reviewed in 54, 55, 56, 57). Underscoring the medical relevance of the IRP/IRE system, mutants of the IRE element of l-ferritin mRNA that lack IRP binding cause hereditary hyperferritinemia–cataract syndrome [58].

IRP1 and IRP2 are ∼60% identical and both are homologous to the mitochondrial TCA cycle enzyme aconitase ACO2 that catalyzes the isomerization of citrate to isocitrate using a cubane iron sulfur cluster (4Fe–4S) as a cofactor. However, only IRP1 displays conservation of the active site, assembles an equivalent 4Fe–4S cluster, and functions as a cytosolic aconitase. RNA-binding and aconitase activity are mutually exclusive. In iron-replete conditions IRP1 ligates the 4Fe–4S cluster and functions as an enzyme, while the cluster is disassembled when iron is scarce and the IRP1 apoprotein binds IREs to its widened cleft [21] (Figure 2C,D). In most tissues a large proportion of the IRP1 pool is in the enzymatically active holoenzyme state [59], leaving a significant reservoir for activation of RNA-binding activity in iron deficiency.

## Moonlighting Central: GAPDH

A second well-characterized example of a protein with dual metabolic and RNA-binding activity is the glycolytic enzyme GAPDH, which converts glyceraldehyde-3-phosphate to d-glycerate-1,3-bisphosphate, generating NADH. In addition to this ‘housekeeping’ role, multiple functions in vesicular trafficking, transcription, DNA repair, telomere maintenance, and cell death have been reported, as reviewed in [60]. Of note, GAPDH is also a part of the interferon γ (IFNγ)-activated inhibitor of translation (GAIT) complex that controls inflammatory mRNA translation in myeloid cells [61]. The heterotetrameric GAIT complex contains the glutamyl-prolyl tRNA synthase (EPRS), NS1-associated protein 1 (NSPA1, also known as SYNCRIP or hnRNPQ), ribosomal protein L13a (L13a), and GAPDH. While NSPA1 is a canonical RBP, EPRS, L13a, and GAPDH need to abandon their regular ‘tasks’ in the multisynthetase complex, the ribosome and glycolysis, respectively, to form the GAIT complex upon phosphorylation of EPRS and L13a by IFNγ-induced kinases [61]. EPRS is the main RNA-binding specificity determinant within the GAIT complex; however, it is unknown whether GAPDH also contributes to the interaction with target RNAs. GAPDH has also been identified as an RBP in its own right, with reported targets ranging from mRNAs, tRNA, rRNA, a ribozyme, and viral RNA (e.g., 20, 62, 63, 64). Multiple reports have focused on GAPDH binding to AU-rich elements (AREs) in the 3′ UTR of numerous mRNAs 20, 62, 65, 66, 67. Competition of RNA binding with NAD^+^ and peptide mapping suggested that the dinucleotide-binding Rossmann fold mediates binding to RNA [20].

**Figure 3:**
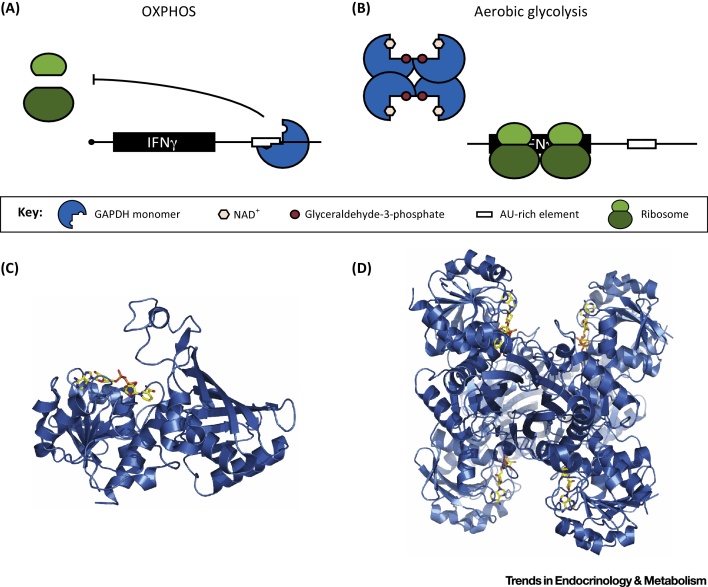
Glyceraldehyde-3-Phosphate Dehydrogenase (GAPDH) moonlights as an RNA-Binding Protein (RBP) in Lymphocytes. (A) GAPDH binds the AU-rich element (ARE) present in the 3′ untranslated region (UTR) of interferon γ (IFNγ) mRNA in resting T cells relying on oxidative phosphorylation (OXPHOS), repressing IFNγ expression. (B) Engaged in glycolysis in activated T cells, GAPDH catalyzes the interconversion between glyceraldehyde-3-phosphate to d-glycerate-1,3-bisphosphate using NAD^+^ as cofactor [25]. (C, D) Ribbon diagram of GAPDH bound to NAD either as a (C) monomer or (D) tetramer (PDB 1ZNQ).

An exciting ‘REM connection’ between gene regulation and metabolism involving GAPDH emerged recently from the study of T cell activation [25] (Figure 3). When T lymphocytes are activated, they switch from oxidative phosphorylation (OXPHOS) to aerobic glycolysis. In cells relying on OXPHOS, translation of IFNγ is repressed by binding of GAPDH to an ARE in the 3′ UTR of the IFNγ mRNA (Figure 3A). This repression is a direct effect of GAPDH as it was preventable by RNAi knockdown, or forced engagement of the enzymatic function of GAPDH by loading T cells with glyceraldehyde-3-phosphate. Following T cell activation and the switch to aerobic glycolysis, GAPDH is no longer active as an RBP bound to IFNγ mRNA and becomes fully engaged in the glycolytic pathway (Figure 3B). Thus, the switch to aerobic glycolysis emerges as a mechanism to antagonize the repression of effector cytokine production by GAPDH, engaging the enzyme in glycolysis rather than RNA binding.

Competition between the enzyme cofactor NAD^+^ and RNA for binding to the same domain on GAPDH could potentially be involved in the above-mentioned effects, as it has been demonstrated that the presence of NAD^+^ or NADH interferes with RNA binding to GAPDH *in vitro* 20, 63, 64, 68, 69. By contrast, the substrate glyceraldehyde-3-phosphate has not shown inhibition of RNA binding, consistent with the RNA interaction not being mediated by the C-terminal substrate-binding region but through the N-terminal Rossmann fold. Enzyme activity is abrogated by the addition of the IFNγ 3′ UTR in a sequence-dependent manner. The RNA also inhibits the assembly of GAPDH monomers into the enzymatically active tetramer (Figure 3C), suggesting that the enzyme binds RNA as a monomer or dimer [19].

While NAD^+^ or NADH interference with RNA binding could be involved in switching between its enzymatic and RNA regulatory functions, GAPDH is also known to be post-translationally modified in various ways, with links to changes in its oligomerization state and subcellular distribution, as reviewed in [60]. *S*-Nitrosylation by nitric oxide at the active site cysteine can trigger GAPDH translocation to the nucleus and activation of its cell death-related functions, as can ADP-ribosylation 70, 71. Intermolecular disulfide bond formation leads to formation of cytoplasmic GAPDH amyloid-like fibrils. Oxidative stress-induced *S*-glutathionylation of the catalytically active cysteine allows the enzyme to participate in shifting metabolic flux from glycolysis to the pentose phosphate pathway 72, 73. Equally, a free sulfhydryl group has been reported to be required for RNA binding [74], and *S*-glutathionylation to block the RNA-binding activity of GAPDH [65]. Thus, alterations in cell state or metabolism could also affect the RNA-binding/enzymatic function of GAPDH via post-translational modifications of the protein.

## Who Affects Whom and How?

**Figure 4:**
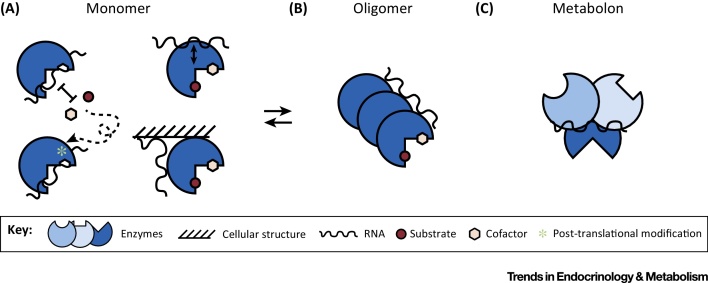
Key Figure: Is RNA Regulated by or the Regulator of Metabolic Enzymes? Several distinct modes of RNA–enzyme interaction can be envisaged

From a conceptual viewpoint, several distinct modes of RNA–enzyme interaction can be envisaged (Figure 4, Key Figure): (i) RNA binding overlaps with the active site and/or cofactor-binding pocket and is in direct competition with substrate or cofactors. If the affinity of the RNA to the enzyme is sufficiently high, this mode of interaction is expected to block catalysis by the enzyme. (ii) The RNA binds to a distinct protein region away from the active site. Such an interaction could either have no effect on catalysis or exert allosteric (positive or negative) effects on the metabolic function of the enzyme. (iii) A special case of the latter scenario is that RNA binding affects interactions of the enzyme with another cellular component, for example, a membrane or other structural element (Figure 4A). (iv) Enzymes often function as homo- or hetero-oligomeric complexes; hence, the interaction with RNA can further bridge between complex subunits or interfere with assembly, when interacting with an oligomerization interface of the enzyme (Figure 4B). And (v) larger assemblies can also exist where enzymes within a pathway are held together by weak interactions to form a ‘**metabolon**’ with superior metabolic flux properties 75, 76, 77. RNA could conceivably bridge between enzymes of a pathway to foster formation of a metabolon (Figure 4C). Interestingly, GAPDH appears to form a higher order complex with other glycolytic enzymes, which was biochemically isolated and shown to be sensitive to RNase digestion [78]. The metabolon concept was first formulated for the TCA cycle [76] and supported by recent work [79]. Similar evidence has been obtained for other pathways such as the enzymes conducting *de novo* purine synthesis, which form purinosomes in the cytoplasm [80]. There is much interest in engineering metabolons for superior performance in biotechnology applications, and indeed a functional metabolon for hydrogen production in live bacteria was designed based on an RNA scaffold [81]. Could RNA-augmented metabolons be a more general occurrence in nature?

Although intuition inspired by existing examples such as aconitase/IRP1 and GAPDH might suggest that an enzyme binding to RNA would control the expression of the RNA (e.g., IRP1 regulating ferritin mRNAs or GAPDH controlling IFNγ mRNA translation), it is important to realize that RNA may also affect the enzyme (e.g., its activity, localization, complex formation, biogenesis, stability, etc.). How would metabolism and metabolites affect this situation? First, metabolites and RNA could affect each other's interactions with the enzyme directly, either through mutually exclusive binding to the same domain or through allosteric effects. Enzymes are often allosterically regulated by metabolites other than their own substrates and thus a given enzyme–RNA interaction might also be regulated by ‘out of left field’ metabolites. Interestingly, even canonical RBPs have been reported to be able to function as metabolite sensors, for example, Musashi-1, which is allosterically inhibited by unsaturated fatty acids [82]. Second, cellular metabolite levels might influence post-translational protein modifications [metabolite-driven post-translational modifications (mPTMs)]. For example, many metabolic enzymes are acetylated and protein acetylation is linked to the cellular levels of both, acetyl-CoA and NAD^+^ [83]. Similar considerations apply to succinyl-CoA and succinylation, malonyl-CoA and malonylation, S-adenosyl methionine and methylation, etc. Thus, metabolism could influence RNA binding to enzymes indirectly, through changes in their PTM status; this could extend the regulatory scope of a metabolite much beyond the enzyme that metabolizes it.

By contrast, RNA itself can act as an effector of the activity of an enzyme. The protein kinase R (PKR) is activated by binding of (pathogen-derived) double-stranded RNA [84]. PKR in a monomeric state is inactive; but the interaction with double-stranded RNA (viral replication intermediaries) triggers its dimerization. As a dimer, PKR is active and can phosphorylate the eukaryotic initiation factor 2α (eIF2α), inducing the inhibition of host cell protein synthesis to prevent viral replication and spread 85, 86, 87. Other examples of RNA-regulated proteins include RIG-I or Toll-like receptor (TLR) 3, TLR7, and TLR8 88, 89. While these examples are taken from the innate immune system and the regulatory RNAs are pathogen-derived, it is perfectly conceivable that host cell genomes could express ‘effector RNAs’ to modulate the functions of RNA-binding enzymes and other RBPs.

These possibilities still await experimental exploration for most of the moonlighting enzymes, as even their physiological RNA partners are not yet known. Nevertheless, the above-mentioned well-studied examples indicate that several, if not all, of the above scenarios deserve consideration for their physiological relevance.

## Concluding Remarks and Future Perspectives

Although cytosolic aconitase has been known for almost a quarter of a century to ‘moonlight’ as an RBP that regulates cellular iron metabolism, it is only now becoming apparent that many metabolic enzymes display RNA-binding activity in living cells. We can currently only speculate about the physiological relevance of this widespread phenomenon, but we point to the urgency of exploring this further to better understand whether and how metabolism and gene regulation might be coupled at this level (see Outstanding Questions). Specifically, we expect that the identification of the RNAs bound by/to different enzymes, and the exploration of their effect on enzymatic function in different cellular contexts will be illuminating. It will also be important to determine how changes in metabolism regulate the interactions between enzymes and RNA, and what the biological consequences of this regulation are.

RNA-binding enzymes could open a whole new chapter in gene regulation and metabolism.

Outstanding Questions What is the physiological function of the RNA-binding activity of so many metabolic enzymes? Do these ‘moonlight’ in the regulation of target (m)RNAs? Does RNA affect aspects of their function as metabolic enzymes?

Which RNAs bind to these enzymes? Identification of these by available technology should shed light on important functional aspects

How do these enzymes bind RNA? What are their RNA-binding domains and do these overlap with regions that are crucial for catalysis or complex formation?

Are enzyme–RNA interactions regulated? Do metabolic signals or metabolites affect these, and if so how is this achieved?

## Figures and Tables

**Table 1:** Examples of Metabolic Enzymes Identified as RBPs in the RNA Interactome Studies

Gene Name	Complete Name	Function	Di/mononucleotide Binding	HeLa RNA Interactome	HEK293 RNA Interactome	mESC RNA Interactome
ADK	Adenylate kinase	AMP biosynthesis	ATP and adenosine	Yes		
ALDH18A1	Delta-1-pyrroline-5-carboxylate synthase	Biosynthesis of proline, ornithine, and arginine	ATP and NADP	Yes		
ALDH6A1	Methylmalonate-semialdehyde dehydrogenase (acylating), mitochondrial	Valine and pyrimidine metabolism	NAD(P)/H	Yes		
ALDOA	Fructose-bisphosphate aldolase A	Glycolysis			Yes	
ASS1	Argininosuccinate synthase	l-Arginine biosynthesis	ATP	Yes		
CCBL2	Kynurenine–oxoglutarate transaminase 3	Transaminase activity for several amino acids			Yes	
CS	Citrate synthase, mitochondrial	TCA cycle			Yes	
DUT	Deoxyuridine 5′-triphosphate nucleotidohydrolase, mitochondrial	Nucleotide metabolism	dUTP	Yes		Yes
ENO1	α-Enolase	Glycolysis		Yes		Yes
FASN	Fatty acid synthase	Fatty acid synthesis	NADP/H	Yes	Yes	
FDPS	Farnesyl pyrophosphate synthase	Formation of farnesyl diphosphate		Yes		
GOT2	Aspartate aminotransferase, mitochondrial	Amino acid metabolism			Yes	
HADHB	Trifunctional enzyme subunit beta, mitochondrial	beta-Oxidation of fatty acids		Yes		
HK2	Hexokinase-2	Glycolysis	ATP			Yes
HSD17B10	3-Hydroxyacyl-CoA dehydrogenase type-2	β-Oxidation at position 17 of androgens and estrogens	NAD/NAD(P)		Yes	
LTA4H	Leukotriene A4 hydrolase	Biosynthesis of leukotriene B4		Yes		
MDH2	Malate dehydrogenase 2, mitochondrial	TCA cycle	NAD/H	Yes	Yes	
NME1	Nucleoside diphosphate kinase A	Synthesis of nucleoside triphosphates	ATP	Yes		
NQO1	NAD(P)H dehydrogenase (quinone) 1	Detoxification pathways and vitamin K-dependent γ-carboxylation of glutamate residues	NAD(P)H	Yes		
PKM2	Pyruvate kinase	Glycolysis	ATP	Yes		Yes
PPP1CC	Serine/threonine–protein phosphatase 1–γ catalytic subunit	Glycogen metabolism, muscle contractility, and protein synthesis			Yes	
SUCLG1	Succinyl-CoA ligase (ADP/GDP-forming) subunit α, mitochondrial	TCA cycle	ATP/GTP	Yes		
TPI1	Triosephosphate isomerase	Glycolysis and gluconeogenesis				Yes

## Glossary

Metabolon: an assembly of several enzymes within a pathway with superior metabolic flux properties.
Moonlighting enzymes: enzymes that carry out a function other than their catalytic activity in metabolism; here, we particularly focus on RNA-binding enzymes.
REM (RNA–enzyme–metabolite) hypothesis: a conceptual framework suggesting that interactions among RNA, enzymes, and metabolites provide regulatory connections between cellular metabolism and gene regulation [17].
RNA interactome capture: an experimental method to purify and identify a near complete compendium of proteins that interact with polyadenylated RNAs in living cells.
